# An improved level set method for vertebra CT image segmentation

**DOI:** 10.1186/1475-925X-12-48

**Published:** 2013-05-28

**Authors:** Juying Huang, Fengzeng Jian, Hao Wu, Haiyun Li

**Affiliations:** 1 Capital Medical University, School of Biomedical Engineering, Beijing 100069, China; 2 Department of Neurosurgery, Xuanwu Hospital Affiliated to Capital Medical University, Beijing 100053, China

**Keywords:** Level set method, Image segmentation, Vertebra CT images

## Abstract

**Background:**

Clinical diagnosis and therapy for the lumbar disc herniation requires accurate vertebra segmentation. The complex anatomical structure and the degenerative deformations of the vertebrae makes its segmentation challenging.

**Methods:**

An improved level set method, namely edge- and region-based level set method (ERBLS), is proposed for vertebra CT images segmentation. By considering the gradient information and local region characteristics of images, the proposed model can efficiently segment images with intensity inhomogeneity and blurry or discontinuous boundaries. To reduce the dependency on manual initialization in many active contour models and for an automatic segmentation, a simple initialization method for the level set function is built, which utilizes the Otsu threshold. In addition, the need of the costly re-initialization procedure is completely eliminated.

**Results:**

Experimental results on both synthetic and real images demonstrated that the proposed ERBLS model is very robust and efficient. Compared with the well-known local binary fitting (LBF) model, our method is much more computationally efficient and much less sensitive to the initial contour. The proposed method has also applied to 56 patient data sets and produced very promising results.

**Conclusions:**

An improved level set method suitable for vertebra CT images segmentation is proposed. It has the flexibility of segmenting the vertebra CT images with blurry or discontinuous edges, internal inhomogeneity and no need of re-initialization.

## Background

Lumber disc herniation is an important cause of lower back pains. Clinical diagnosis and therapy for the lumbar disc herniation requires the knowledge of the stress and strain throughout the lumbar region [1]. The finite element method based on medical images is able to analyze the biomedical characteristic of lumbar in the compression. We are sure that accurate 2D vertebra segmentation will help us reconstruct 3D vertebra geometric model because 3D vertebra segmentation modeling is fundamentally performed based on a set of axial slices. The understanding of geometrical information about the normal anatomy and the degenerative bony deformations of the spine necessitates vertebra CT image segmentation for the clinical diagnosis and the preoperative planning of spinal diseases.

There are several proposed approaches in the literature for vertebra segmentation. Statistical shape models (SSMs) [2,3], which generated mean shapes using their own shape parameters by Fourier and wavelet descriptors, used shape constraints to overcome ambiguous boundary information. Active shape models (ASMs) [4] was a kind of SSMs that iteratively searched a boundary while maintaining shape constraints. Although SSMs and ASMs could overcome an ambiguous boundary problem, they could not converge into an unusual shape or represent small variations in a boundary. Active appearance models (AAMs) [5] which combined appearance information and shape constraints, could provide better robust results than ASMs in many medical segmentation applications. However, its application to vertebra segmentation was difficult because the texture patterns of vertebra bodies are different among patients. A deformable spine model [6] using landmarks exploited shape information and gray-level inhomogeneities using necklace and string models. The necklace model captured variations in vertebra structures while the string model represented spinal curvatures. However the deformable spine model could be trapped into a local minimum and failed to segment abnormal vertebra. Yao J [7] segmented a vertebra by fitting a four-part vertebra model, but the segmentation could not separate the vertebra region into composing vertebra bones, where a spinous process belonging to the upper vertebra exists with a transverse process pertaining to the current vertebra. Hong S [8] proposed the concept of localized priors which guided the level set to avoid leakage and local minimum at the places where most necessary, then segmented the completed individual vertebras from the complex neighboring structures. Multiple level set methods [9,10] were used to extract only vertebra bodies but not to segment spinous parts. Kim Y [11] presented a fully automatic vertebra segmentation method using 3D deformable fences (3DDF) for 3D CT images, which extracted 2D curve with a deformable model that utilized 3D valley information and was expanded to form a 3D surface. However, it was not robust to segment the vertebral images with weak valley information occurring in abnormal cases. Klinder T [12] first used various kinds of models, such as shape, gradient, and appearance information, and applied 3D deformable model approach to segment the vertebra CT images. Although they achieved very competitive identification rates for vertebrae, their algorithm depends heavily on spatial registration of the deformable model, which is computationally very expensive. Interactive tools for spine segmentation [13] were developed to achieve more accurate results. Although the interactive method provides protocols for segmentation, it still required a laborious manual process. Poay et al. [14] focused on 3D segmentation firstly introducing willmore flow into the level set method (WFLS). The framework incorporated prior shape knowledge through the KDE and local geometrical features by introducing Willmore flow into the level set segmentation and obtained good 3D segmentation results of normal spinal vertebra images.

The shape of the vertebra exhibits complicated topological characteristics. The boundaries in vertebra CT images are ambiguous and discontinuous, while the intensity in vertebra CT images is highly inhomogeneous. The complex shape and inhomogeneous intensity in the vertebra CT images makes its segmentation challenging. In this paper, we developed an improved level set model to achieve a 2D vertebra extraction method. By introducing the edge detection function (*edf*) and region detection function (*rdf*) into the proposed model, the images with ambiguous or discontinuous boundary and intensity inhomogenity can be effectively segmented. At the same time, we automatically initialize the level set function by Otsu threshold, thus roughly obtain the regions of interest and multiple initial curves. The curves evolve stably and quickly according to the evolution equation, with its zero level set curves converged to the exact boundary of regions of interest. The algorithm can obtain the accurate segmentation results not only when the internal intensity of vertebra CT images is inhomogeneous, but also when the boundary in CT images is ambiguous or discontinuous. Besides, the algorithm needs no costly re-initialization because of the regularization term, which improves the segmentation speed greatly.

This paper is organized as follows. We briefly review some vertebra segmentation methods and well-known level set methods in "Background". Our edge- and region- based level set (ERBLS) model is presented in "Methods". In "Results", our proposed model is validated by some experiments on synthetic and real images. In "Discussion", we discussed our proposed method and compared our segmentation results with those of 3DDF method [12] and WFLS method [14]. Finally, some conclusive remarks are included in "Conclusion".

### The related methods

Level set method was developed by Osher and Sethian in 1988 [15], which was an effective method of contour evolution. It utilizes dynamic variational boundaries for image segmentation and can be categorized into two types: edge-based models [16] and region-based models [17].

Early level set methods [18-22] mostly belong to edge-based models, which mainly use image gradient to construct an edge detecting function to stop the contour evolution on the object boundary. The popular formulation for level set segmentation is [23]

(1) $$\frac{\partial \phi }{\partial t}=g\left|\nabla \phi \right|\left(\text{div}\left(\frac{\nabla \phi }{\left|\nabla \phi \right|}\right)+\nu \right)$$

where div(∇*ϕ*/|∇*ϕ*|) approximates mean curvature, *ν* is a balloon force and *φ* is the level set function. The function *g* is image gradient, namely an edge detecting function (*edf*(*I*)), which is defined as

(2) $$\mathrm{edf}\left(I\right)=\frac{1}{1+\left|\nabla G_{\sigma }*I\right|}$$

where *G*_*σ*_ * *I* stands for the convolution of the image *I* with a smoothing Gaussian kernel *G*_*σ*_. The range of *edf* (*I*) is between 0 and 1. This edge detector has low values close to 0 at the object boundary, and high value closes to 1 at homogenous background.

The regularity of *φ* is very important for stable evolution and accurate computation in level set methods. A common way to reinitialize *φ* is to set |∇*ϕ*| = 1 before the curve deviates from the level set function, so that the curve can evolve stably and accurate segmentation results can be obtained. However, the re-initialization is very complicated and may bring some side effects, e.g., the evolving level set function can deviate remarkably from the signed distance function with a few iterations, especially when the time step chosen is not small enough. In order to overcome the problem, a fast level set formulation was proposed [24]

(3) $$\frac{\partial \phi }{\partial t}=\mathrm{\mu P}\left(\phi \right)+\eta \left(g,\phi \right)$$

where *μ*>0 is a parameter controlling the strength with which the deviation of *φ* from a signed distance function is penalized. The first term *P* (*φ*) penalizes the deviation of *φ* from a signed distance function during its evolution and is defined as the following:

(4) $$P\left(\phi \right)=\Delta \phi -\mathrm{div}\left(\frac{\nabla \phi }{\left|\nabla \phi \right|}\right)$$

The second term *η*(*g*,*φ*) incorporates the image gradient information by

(5) $$\eta \left(g,\phi \right)=\lambda \delta \left(\phi \right)\mathrm{div}\left(g\frac{\nabla \phi }{\left|\nabla \phi \right|}\right)+\mathrm{\nu g\delta}\left(\phi \right)$$

where δ(*φ*) denotes the Dirac function. The parameters *μ*, *λ* and *ν* control the individual contributions of these terms.

In essence, the term *η*(*g*,*φ*) attracts *φ* towards the variational boundary, which is similar to the standard level set method. The penalty term *P*(*φ*) eliminates the computationally expensive re-initialization for signed distance functions. This modification leads to a fast level set algorithm for image segmentation. However, the edge-based level set method only uses the edge detecting function to stop evolving curves, which results in the active contours leaking out the ideal contours when the edges are ambiguous.

To solve the boundary leaking problem, Zhang et al. [25] proposed a region-based active contour model with a region-based signed pressure force (SPF) function which can efficiently stop the contours at weak or blurred edges. This model only uses the image statistical information of the entire region inside and outside the contour, and is unable to successfully segment images with intensity inhomogeneity.

To overcome the difficulty caused by intensity inhomogeneities, Li et al. proposed the local binary fitting (LBF) model [26,27], which makes use of the local intensity information. In the LBF model, two spatially varying fitting functions *f*_1_(*x*) and *f*_2_(*x*) are introduced to approximate the local intensities on the two sides of the contour, and for a given point *x*∈Ω, the local intensity fitting formulation is:

(6) $$\frac{\partial \phi }{\partial t}=\delta \left(\phi \right)\left(\mu \mathrm{div}\left(\frac{\nabla \phi }{\left|\nabla \phi \right|}\right)-\lambda_{1}e_{1}+\lambda_{2}e_{2}\right)+\nu \left(\nabla^{2}\phi -\mathrm{div}\left(\frac{\nabla \phi }{\left|\nabla \phi \right|}\right)\right)$$

Where λ_1_ and λ_2_ are positive constant, and *e*_1_ and *e*_2_ are the functions as the following

(7) e=1∫ΩKy-xσIx-f1yd2ye=2∫ΩKσy-xIx-f2yd2y

Where *K*_*σ*_(*y* − *x*)is a Gaussian kernel function, and *f*_1_(*x*) and *f*_2_(*x*) are two values that approximate image intensities inside and outside contour C, respectively.

(8) $$\left\{\begin{array}{l} f_{1}\left(x\right)=\frac{K_{\sigma }\left(x\right)\left[H\left(\phi \left(x\right)\right)I\left(x\right)\right]}{K_{\sigma }\left(x\right)\left[H\left(\phi \left(x\right)\right)\right]}\\ f_{2}\left(x\right)=\frac{K_{\sigma }\left(x\right)\left[\left(1-H\left(\phi \left(x\right)\right)\right)I\left(x\right)\right]}{K_{\sigma }\left(x\right)\left[1-H\left(\phi \left(x\right)\right)\right]} \end{array}\right)$$

The LBF model is able to obtain desirable segmentation sometimes in the presence of intensity inhomogeneity. At the same time, the time-consuming re-initialization is avoided. However, the computational cost is still very high, which is pointed out by Zhang et al. [28]. In addition, the LBF model is sensitive to initialization to some extent [29], which limits its practical applications. Recently, Liu et al. [30] proposed LRCV model, which have similar capability of handling intensity inhomogeneity as LBF model.

## Methods

In this section, we present and discuss in detail the proposed edge- and region-based level set model (ERBLS). For a point *x* ∈ *Ω*, its intensity can be approximated by a weighted average of the image intensity *I*(*y*) where *y* is the neighborhood of *x*. Then region detecting function (*rdf*(*I*(*x*))) can be defined by the following:

(9) $$\mathrm{rdf}\left(I\left(x\right)\right)=\frac{g_{\sigma }\left(x-y\right)*I\left(x\right)-\frac{c_{1}+c_{2}}{2}}{\max\left(\left|g_{\sigma }\left(x-y\right)*I\left(x\right)-\frac{c_{1}+c_{2}}{2}\right|\right)},\left(x\in \Omega \right)$$

*c*_1_and*c*_2_are given by:

(10) $$\begin{array}{l} c_{1}\left(x\right)=\frac{\int_{\Omega }g_{\sigma }\left(x-y\right)*I\left(y\right)\cdot H\left(\varphi \left(y\right)\right)dy}{\int_{\Omega }g_{\sigma }\left(x-y\right)*H\left(\varphi \left(y\right)\right)dy}\\ c_{2}\left(x\right)=\frac{\int_{\Omega }g_{\sigma }\left(x-y\right)*I\left(y\right)\cdot \left(1-H\left(\varphi \left(y\right)\right)\right)dy}{\int_{\Omega }g_{\sigma }\left(x-y\right)*\left(1-H\left(\varphi \left(y\right)\right)\right)dy} \end{array}$$

where *g*_*σ*_(*x* − *y*)is a Gaussian kernel function with an averaging filter of *k* × *k*size and can be considered as the weight assigned to each intensity *I*(*y*) at *y*. Due to the location property of the kernel function *g*_*σ*_ (*x-y*), the contribution of the intensity *I*(*y*) to *c*_1_(*x*) and *c*_2_(*x*) decrease and approach to zero as the point *y* goes away from the center point *x*. Therefore, *c*_1_(*x*) and *c*_2_(*x*) are determined by the intensities of the points in the neighborhood of the point *x*. Then the region detection function (*rdf*) is also dominated by the intensities of the points in the neighborhood of the point *x*.

The energy functional consists of three parts: edge information term*βE*^*E*^, local region information term *γE*^*LR*^ and regularization term*E*^*R*^, which is defined as following:

(11) Eφ=βEE+γELR+ER=β∫Ωedfxδφ∇φdx+γ∫Ω(rdfIxH−φdx+∫Ω12∇φ−1d2x

where *β* and *γ* are fixed constants.

Fixing *c*_1_(*x*) and *c*_2_(*x*), we minimize Equation (11) and obtain the corresponding variational level set formulation as follows:

(12) $$\frac{\partial \varphi }{\partial t}=\beta \delta \left(\varphi \right)\mathrm{div}\left(\mathrm{edf}\left(x\right)\frac{\nabla \varphi }{\left|\nabla \varphi \right|}\right)+\gamma (\mathrm{rdf}\left(I\left(x\right)\right)\delta \left(\varphi \right)+\frac{1}{2}\left[\Delta \varphi -\mathrm{div}\left(\frac{\nabla \varphi }{\left|\nabla \varphi \right|}\right)\right]$$

It is obvious that the above equation has the merits of both edge-based models and region-based models. When the contour is far away from object boundaries, the force from the local region intensity information is dominant and has a certain capture range. When the contour is close to the object boundaries, the force from the gradient information becomes dominant, which attracts the contours and finally stop the contours at the object boundaries. The technique of using local region information can improve the robustness to the initialization of contours. When the boundary is blurred or discontinuous, the interference from the local intensity force is able to result in contours’ stopping at the real object boundary. Furthermore, due to the region stopping function making use of local region information, the ERBLS model is able to provide desirable segmentation results even in the presence of the images with intensity inhomogeneity. Besides, our method introduces a new penalizing energy to the regularization term, therefore the computational cost is heavily decreased.

In order to effectively calculate the level set function *φ*, the Heaviside function *H*(*φ*) here is normalized as

(13) $$H\left(z\right)=\frac{1}{2}\left(1+\frac{2}{\pi }\arctan\left(\frac{z}{\epsilon }\right)\right)$$

(14) $$\delta_{\epsilon }\left(z\right)={H^{\prime }}_{\epsilon }\left(z\right)=\frac{1}{\pi }\frac{\epsilon }{\epsilon^{2}+z^{2}}$$

In the proposed ERBLS model, the main computational cost comes from computing *c*_1_(*x*) and *c*_2_(*x*) in Equation (10). At the first sight, there are four convolutions to compute *c*_1_(*x*) and *c*_2_(*x*). It can be noticed that the expression can be rewritten to the combination of the four convolutions: ∫ _*Ω*_*g*_*σ*_(*x* − *y*)d*y*, ∫ _*Ω*_*g*_*σ*_(*x* − *y*)*H*(*ϕ*(*y*))d*y*, ∫ _*Ω*_*g*_*σ*_(*x* − *y*)*I*(*y*)d*y* and ∫ _*Ω*_*g*_*σ*_(*x* − *y*)(*I*(*y*)*H*(*ϕ*(*y*)))d*y*. Because the two convolutions ∫ _*Ω*_*g*_*σ*_(*x* − *y*)d*y* and ∫ _*Ω*_*g*_*σ*_(*x* − *y*)*I*(*y*)d*y* can be computed only once before the iterations, the terms ∫ _*Ω*_*g*_*σ*_(*x* − *y*)d*y* and ∫ _*Ω*_*g*_*σ*_(*x* − *y*)*I*(*y*)d*y* do not depend on the evolution of level set function*φ*. Therefore there are only two convolutions ∫ _*Ω*_*g*_*σ*_(*x* − *y*)*H*(*ϕ*(*y*))d*y* and ∫ _*Ω*_*g*_*σ*_(*x* − *y*)(*I*(*y*)*H*(*ϕ*(*y*)))d*y* to be computed at each iteration. In comparison, there are at least four convolutions in the LBF model [27]. As a result, the computational cost of the ERBLS model is about half that of the LBF model for each iteration.

### The region detecting function (*rdf* (*I*(*x*)))

The implication of Equation (9) can be explained as follows. Suppose that the intensities inside and outside the object are homogeneous. It is intuitive that min (*g*_*σ*_(*x* − *y*) * *I*(*x*)) < *c*_1_, *c*_2_ < max (*g*_*σ*_(*x* − *y*) * *I*(*x*)) and the equal signs cannot be obtained simultaneously because $\min\left(g_{\sigma }\left(x-y\right)*I\left(x\right)\right)<\frac{c_{1}+c_{2}}{2}<\max\left(g_{\sigma }\left(x-y\right)*I\left(x\right)\right)$ wherever the contour is. The signs of the region detecting function *rdf*(*I*(*x*)) inside and outside the object are opposite. The signs of the *rdf*(*I*(*x*)) inside the object are negative and those outside the object are positive. The curve of the level set function expands when *rdf*(*I*(*x*)) is negative, and contracts when *rdf*(*I*(*x*)) is positive. Besides, the larger the magnitude of *rdf*(*I*(*x*)), the faster the level set evolves. It is obviously advantageous to make the level set function evolve faster, if contours are far away from the real boundary. On the contrary, the evolution velocity of the level set function should have been slowed down once contours approach the boundary. Moreover, the level set function should alter its direction of movement automatically, while passing through the boundary of interest.

**Figure 1:**
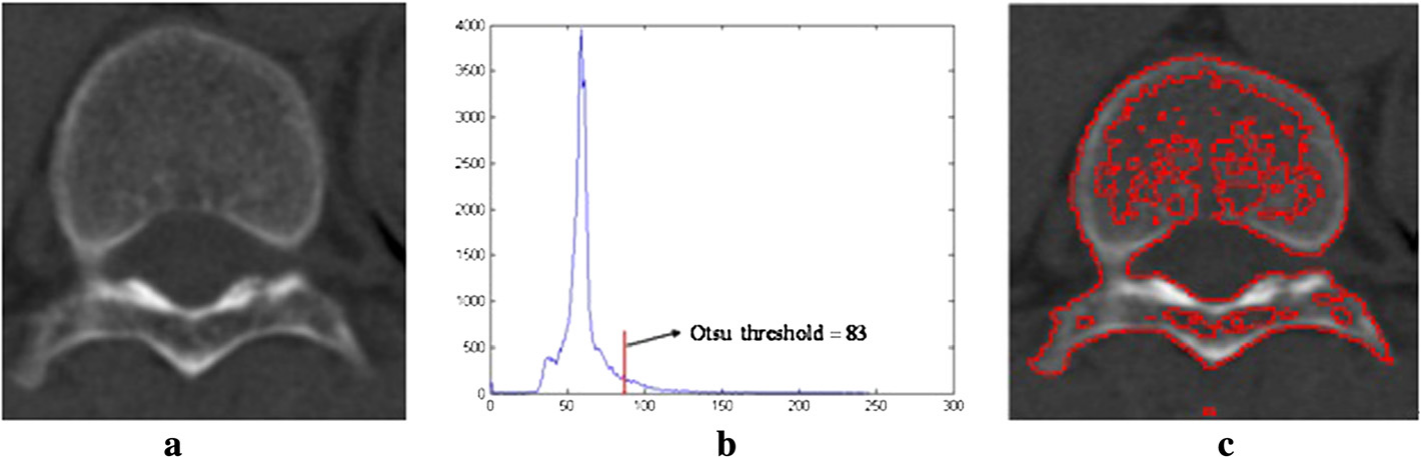
**Signs of the *rdf *(*I*(*x*)) inside and outside the object are opposite.**

### Automatic initialization by Otsu threshold

Thresholding is an essential region-based image segmentation technique that is particularly useful for separating objects from the background [31-33]. In our proposed method, an optimal threshold can be obtained automatically by Otsu algorithm implemented in Matlab 7.0. Otsu’s method could be used to perform histogram shape-based image thresholding. The Otsu’s method assumes that the image has two classes of pixels or bi-modal histogram and then intra-class variance is minimal and the two classes are separated by the optimum threshold [34]. The vertebra images were assumed to have the two classes, one is object and the other is background. According to Otsu’s method, the Otsu threshold of a vertebrae CT image can be obtained automatically. Figure 1(a) is original image, Figure 1(b) is histogram of the original image, the Otsu threshold of the original image is 83, and Figure 1(c) is the initialized image by Otsu threshold.

**Figure 2:**
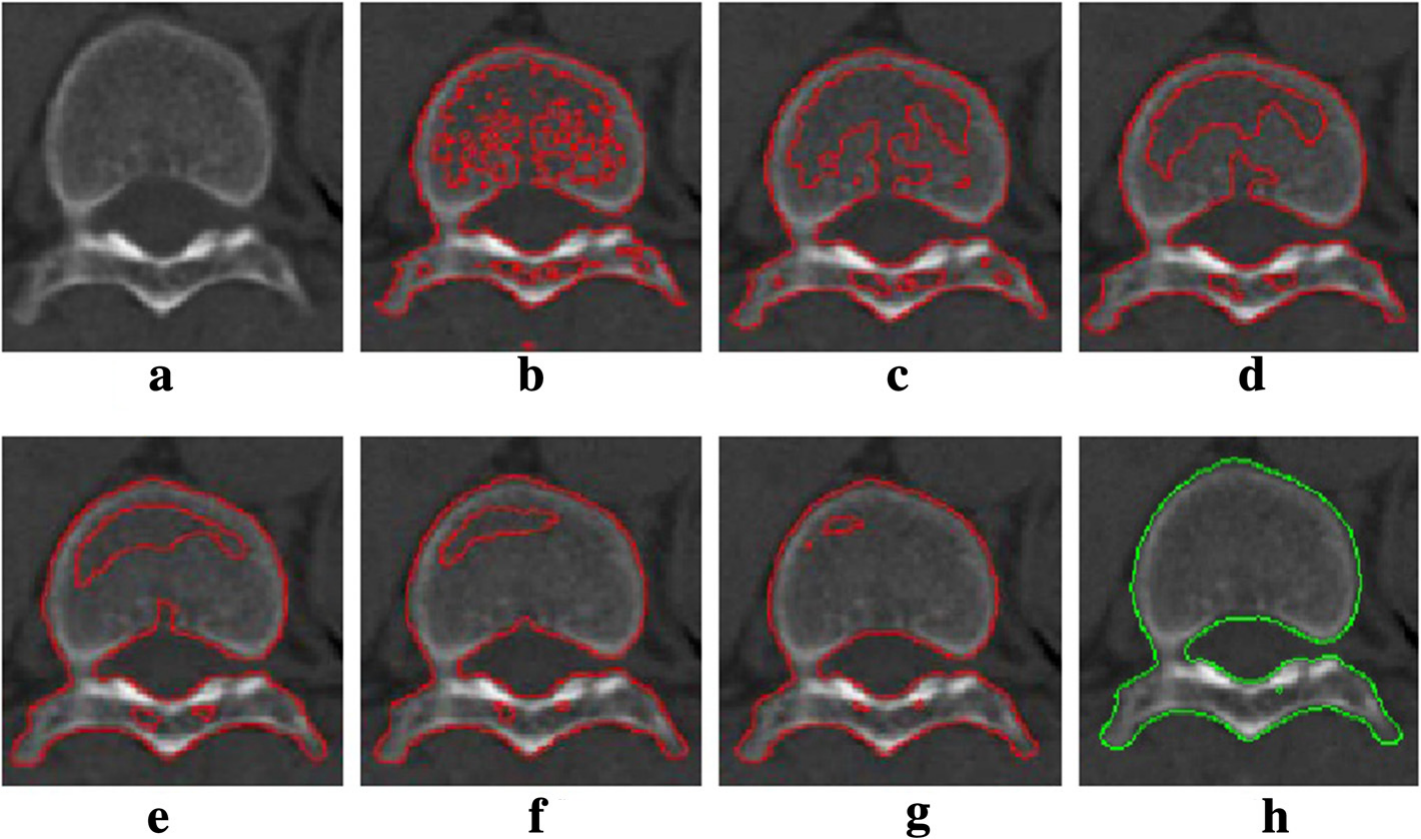
**Automatic initialization by Otsu threshold. (a)** original image; **(b)** histogram; **(c)** initialized image by Otsu threshold.

After the level set function is initialized by the optimal threshold obtained automatically, the regions of interest are roughly and automatically delineated [35]. Then we can use these regions to construct the initial level set function, which also affects computational efficiency. The initial curves (level set function) will evolve stably according to the evolution equation, with its zero level set curves convergence to the exact boundary of the region of interest. Figure 2 shows the segmentation process of a vertebral image using our improved method. The objective is to extract the vertebra which appears brighter than the background in the image, as shown in Figure 2(a). The initial contours are obtained by Otsu threshold, as shown in Figure 2(b). As shown in Figure 2(c) (d) (e) (f) (g) (h), the evolving curves continue to expand, contract, split or merge, then the vertebra is successfully segmented at the 180th (Figure 2(h)). During the process, controlled by the region detecting function and the edge detecting function, the contours expand, contract, split or merge, and stop at the desired places. As a result, accurate segmentation is obtained.

**Figure 3:**
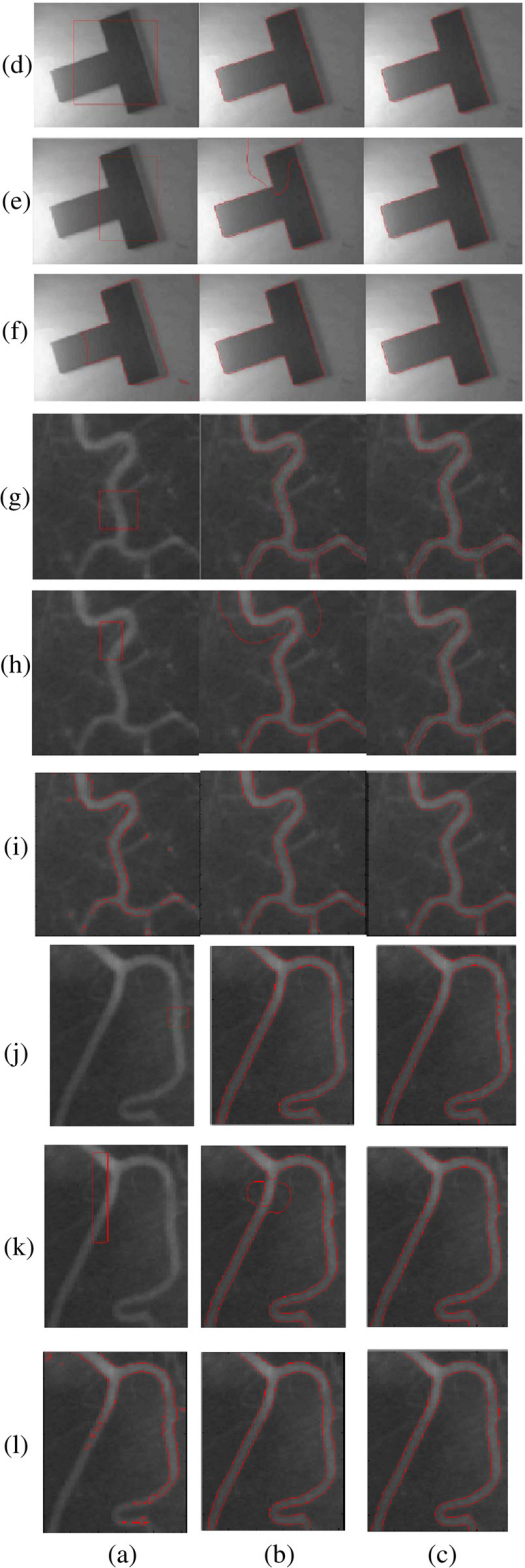
**Illustration of segmentation procedure. (a)** original image, **(b)** initial contours, **(c)** 10th iteration; **(d)** 30th iteration; **(e)** 60th iteration; **(f)** 100th iteration; **(g)** 140th iteration; **(h)** final segmentation result at 180th iteration. Size=482×423.

With the above procedures, the initialization of level set function by Otsu threshold can be completely automatic without any human interfaces. The segmentation result can then be taken as the initial contours for the evolution of the ERBLS model.

## Results

The clinical image data set was acquired by the Department of Neurosurgery of Beijing Xuanwu Hospital Affiliated to Capital Medical University in China in compliance with the Helsinki Declaration approved by Guojun Zhang. The data set consists of 56 CT images of intervertebral disc protrusion images of patients aged 18 to 66. The patients are carefully selected by radiologists to form a representative group. These images are acquired from 64-detector row Siemens CT System. The in-plane resolution for these images is 1 mm with slice thickness of 1.5 mm. Original images have fixed sizes of 512×512 and the total number of vertebrae is 293. The ground truths are delineated by clinical experts. Our algorithm is implemented in Matlab 7.0 on 2.79-GHz Intel Pentium IV PC. Unless otherwise specified, we used the following parameters in our model: σ=3.0, ϵ=1.0, *β*=5.0, γ=2.0, time increment Δt=1.0.

The proposed method has been tested with synthetic and real images. First we used the LBF model [27] and the proposed ERBLS model to segment one synthetic image and two blood images with intensity inhomogeneity in Figure 3. As we discussed in "Methods", the LBF model usually needs to perform four convolution operations at each iteration and is sensitive to the selection of governing parameters and the location of initial contour. We tried many times and selected the best governing parameters *μ* = 0.001 × 255^2^ (the length controlling parameter), sigma=5/5/3.5 (the standard deviation of Gaussian kernel for two images). In Figure 3, column (a) shows various initial contours; column (b) and column (c) are the segmentation results by the LBF model and the ERBLS model. The initial contours in Rows (d), (e), (g), (h) (j) and (k) are generated manually. The initial contours in row (f) (i) and (l) are obtained by Otsu threshold. For some initial contours, as shown in Rows (e) (h) and (k), the LBF model fails. For all initial contours, the right segmentation results can be obtained from the ERBLS model. The numbers of iteration and CPU running time of the two models are listed in Table 1. It can be seen from Table 1 that iteration numbers and processing time for the ERBLS model are both less than that of LBF model for all three image segmentation. Considering that the parameters and initial contours of the LBF model are selected elaborately, so the ERBLS model is proved to be more efficient in segmenting the image with the intensity inhomogeneity.

**Table 1:** **Iteration number and processing time for the LBF model and proposed ERBLS model in segmenting the images in Figure**3

	**LBF model**		**ERBLS model**	
	**Iteration numbers**	**CPU time (s)**	**Iteration numbers**	**CPU time (s)**
Row (d)	300	26.172	220	6.0625
Row (e)	300	26.563	210	7.1285
Row (f)	160	10.016	100	2.3280
Row (g)	300	3.734	230	0.8212
Row (h)	330	4.5720	300	1.0680
Row (i)	150	2.9531	60	0.8125
Row (j)	300	6.2969	260	3.5469
Row (k)	400	11.7969	260	3.728
Row (l)	220	5.2594	100	1.3750

**Figure 4:**
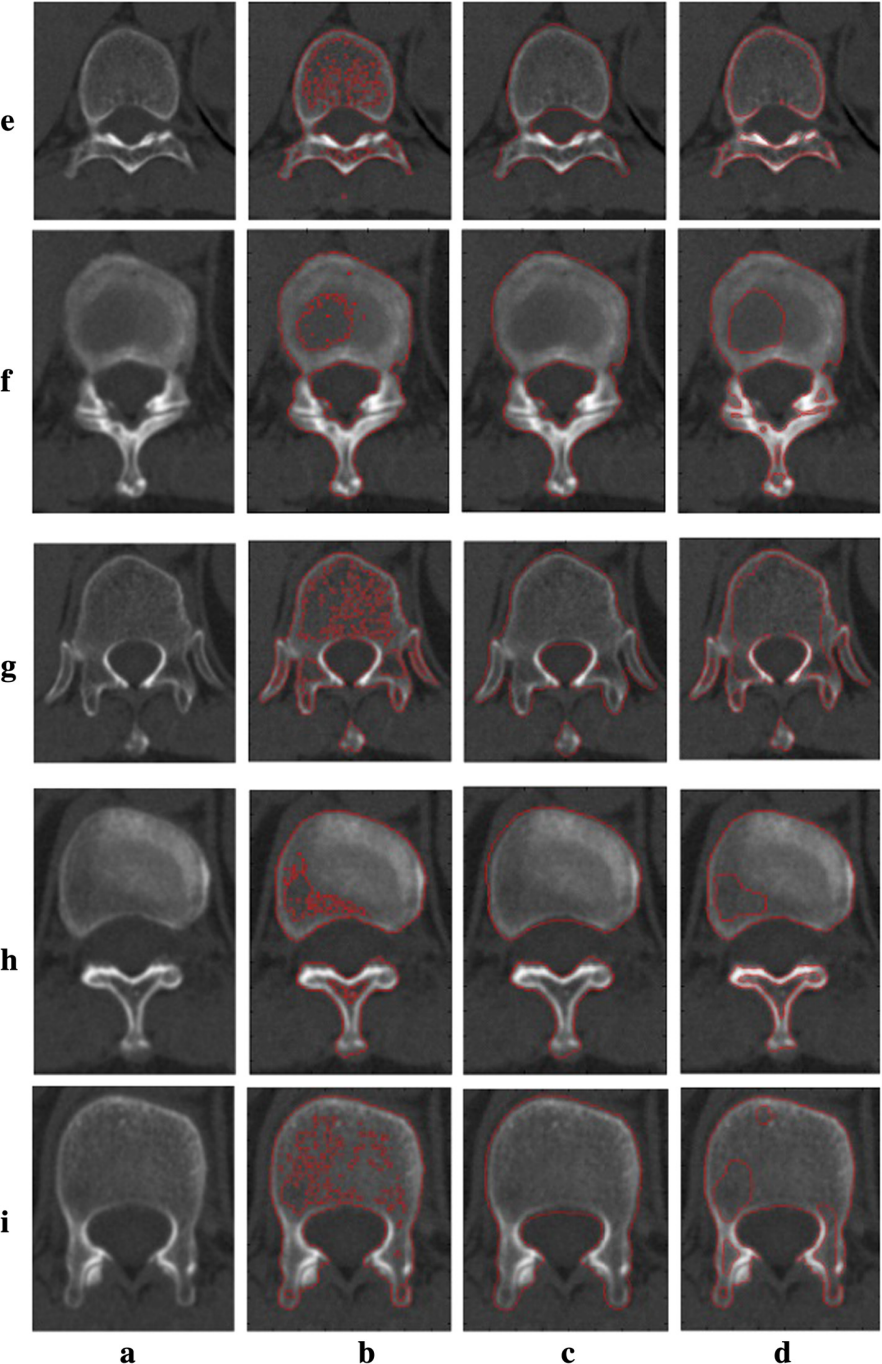
**Comparisons of the LBF model and the proposed ERBLS model on segmenting synthetic and two real blood vessel images with intensity inhomogeneity.** Column **(a)**: initial contours. The initial contours in row **(f) (i)** and **(l)** are obtained by Otsu threshold. Column **(b)**: final segmentation results using the LBF model. Column **(c)**: final segmentation results using our proposed ERBLS model. Size=127×96, 111×110, 103×131.

We show the segmentation results on vertebra CT images with intensity inhomogeneity which boundaries are somewhat ambiguous and discontinuous in Figure 4. Refer to Figure 4, column (a) is original images; column (b) is the initial contours obtained by using Otsu threshold columns; (c) and (d) are the segmentation results by the ERBLS model and the LBF model, respectively. Shown in Figure 4, we can see that for the images with ambiguous, discontinuous boundary and intensity inhomogeneity, the LBF model cannot obtain the right segmentation results. In our improved method, the initial curves evolve according to Equation (9), even if the boundaries in the images are obscure and discontinuous, the ideal segmentation results are obtained. The numbers of iteration and CPU running time of the two models are listed in Table 2. This illustrates that the proposed ERBSL is more robust than the LBF model in segmenting vertebral CT images.

**Table 2:** **Iteration number and processing time for the LBF model and the proposed ERBLS model in segmenting the images in Figure**4

	**LBF model**		**ERBLS model**	
	**Iteration numbers**	**CPU time (s)**	**Iteration numbers**	**CPU time (s)**
Row (e)	420	20.2813	180	2.9375
Row (f)	300	9.5321	150	0.4844
Row (g)	400	19.5000	200	2.2813
Row (h)	380	27.0625	150	2.0625
Row (i)	300	14.625	140	0.7965

**Figure 5:**
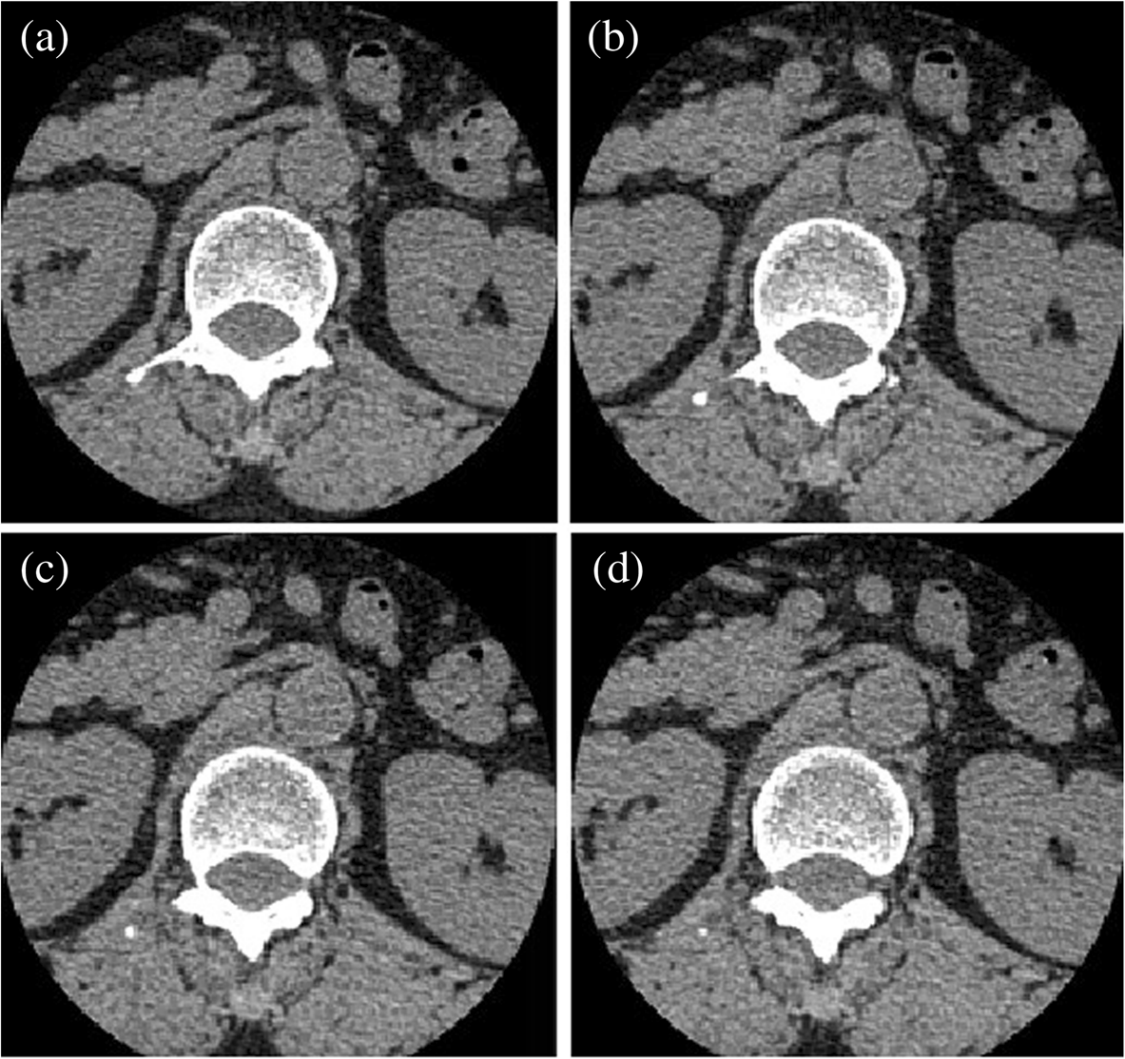
**Comparisons of the LBF model and the proposed ERBLS model on segmenting five vertebra CT images with the intensity inhomogeneity.** Column **(a)** original images; Column **(b)** initial contours by using Otsu threshold; Column **(c)**: final segmentation results using our proposed ERBLS model; Column **(d)**: final segmentation results using the LBF model.

## Discussion

In order to further evaluate our segmentation algorithm, we reconstructed 3D vertebra images based on 2D segmentation results by using our proposed method. The proposed method has been applied to 56 patient data sets and the segmentation results are compared with those of 3D deformable fences method (3DDF) [11] and introducing willmore flow into level set segmentation (WFLS) [14].

**Figure 6:**
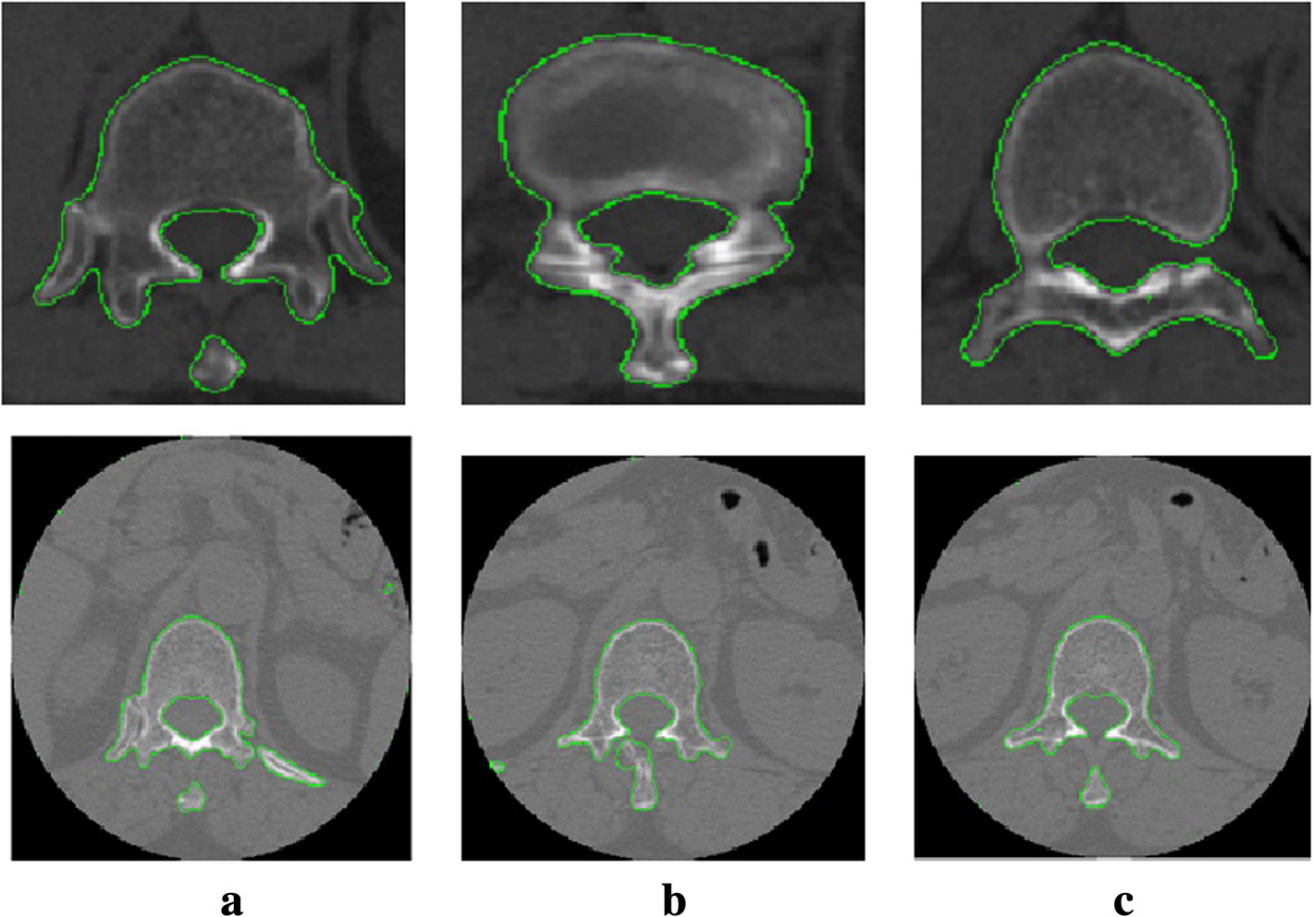
**Neighboring slices are similar in 2D CT image data set. (a)** image slice 31; **(b)** image slice 32;**(c)** image slice 33; **(d)** image slice 34.

**Figure 7:**
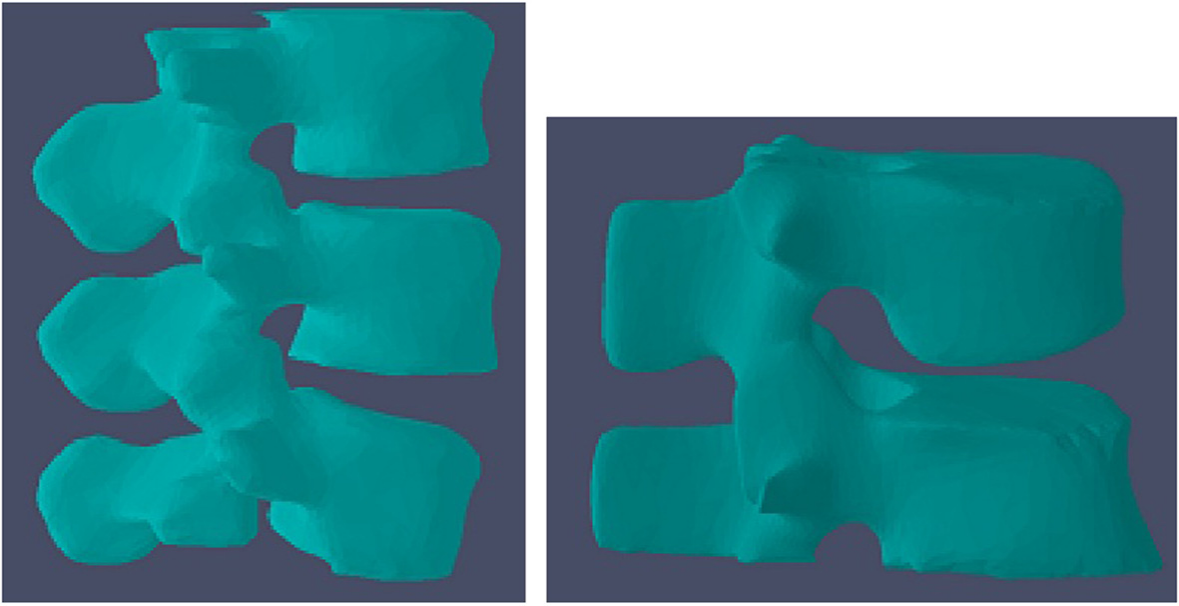
**2D segmentation results.** Columns **(a) (b) (c)** are segmentation results of 2D CT slices.

Because the vertebral boundaries of neighboring slices are usually similar (shown in Figure 5), the evolving contours of current slice provide a good initialization for the neighboring ones, hence we use the current slice to initialize the contour in adjacent slice [36]. This can save computation and improve the efficiency and accuracy of the results. For example, the segmentation result of slice 31 is used to initialize the slice 32, thus vertebral regions and non-vertebral regions are roughly obtained. We then compute *c*_1_、*c*_2_ and region detecting function *rdf*(*I*(*x*)) as described in "Methods". After iteration, the entire volumetric image is processed. Segmentation results for 2D slices are shown in Figure 6 and the reconstructed 3D images based on the 2D segmentation results are shown in Figure 7.

Segmentation accuracy is very important for clinical image diagnosis. We adopted the Dice similarity coefficient (DSC) [37] and Hausdorff distance (HD) [38] to evaluate the segmentation accuracy. The manual segmentation results by clinical experts are considered as ground truth. DSC measures the spatial overlap between two segmentations, HD measures the relative differences between boundaries of the segmented objects. The DSC is formulated as

(15) $$D\left(\Omega_{O},\Omega_{G}\right)=\frac{2\left|\Omega_{O}\cap \Omega_{G}\right|}{\left|\Omega_{O}\right|+\left|\Omega_{G}\right|}$$

where $\left|\Omega \right|$ and $\left|\Omega \right|$ represent the volumes of segmented object Ω and the ground-truth Ω respectively. The measurement (varies from 0 to 1) indicates the correspondence between two volumes, i.e., 0 indicates the two volumes do not overlap and 1 shows they are perfectly matched.

On the other hand, the HD is the maximum distance of a set to the nearest point in the other set, defined as

(16) $$d_{H}\left(A,B\right)=\max\left\{\underset{a\in Ab\in B}{\sup\inf d\left(a,b\right)},\underset{b\in Ba\in A}{\sup\inf d\left(a,b\right)}\right\}$$

where A and B are the boundaries of two different segmented volumes, respectively. It measures the distance between the farthest point of a set to the nearest point of the other. The measurement (varies from 0 to ∞ theoretically) represents the difference between two closed surfaces, e.g., 0 shows that both volumes share exactly the same boundaries, and larger HD values indicates larger distances between the boundaries. In summary, a high DSC and a low HD are desirable for good segmentation.

Results for 293 vertebrae from 56 patient data sets are summarized in Table 3. As can be seen, our proposed method produces good segmentation results (DSC 0.94±0.02, HD 10.06±1.71 mm) compared with 3D deformable fence method (DSC 0.80±0.02, HD 16.23±2.13 mm) and introducing willflow into level set method (DSC 0.88±0.03, HD 14.03±2.18 mm). In our improved method, the initial curves evolve according to Equation (9), even if the boundaries in the images are obscure and discontinuous, the ideal 2D segmentation results are obtained. The 3D vertebra images are reconstructed based on the ideal 2D segmentation results, therefore, the DSC value of the 3D segmentation results obtained by our proposed method is large and the HD value of that is low.

**Table 3:** Average DSC and HD (mm) with standard deviation for segmentation of vertebra CT images using our ERBLS method, 3DDF method and WFLS method

	**ERBLS method**	**3DDF method**	**WFLS method**
DSC	0.94±0.02	0.80±0.02	0.88±0.03
HD (mm)	10.06±1.71	16.23±2.13	14.03±2.18

## Conclusion

We have described an edge- and region-based level set method for accurate segmentation of vertebra CT images. The ERBLS model can efficiently segment the images with intensity inhomogenity and blurry or discontinuous boundaries by employing the image gradient information and the local image information. Meanwhile, the level set function is automatically initialized by Otsu threshold, which segmentation result is taken as the initial contours of the EBRLS model. Experimental results on both synthetic and real images demonstrated that the proposed ERBLS model is very robust and efficient. Compared with the well-known local binary fitting (LBF) model, the ERBLS model is not only much more computationally efficient and but also much less sensitive to the initial contours. The proposed method has also applied to 56 patient data sets and has produced very promising results.

## Competing interests

The authors declare that they have no competing interests.

## Authors’ contributions

JH worked on the algorithm design and implementation, and wrote the paper; FJ and HW provided CT images and clinical instruction. HL contributed discussions and suggestions throughout this project, including the manuscript writing. All authors read and approved the final manuscript.

## Authors’ information

About the Author—Juying Huang, PhD, is working at the medical physics education and research work. Her current research interests are medical image processing, CT image reconstruction, medical system modelling and computing.

About the Author—Fengzeng Jian, PhD, Chief physician, is working at clinical and research work in the spine and skull base surgery.

About the Author—Hao Wu, PhD, associate Chief physician, is mainly working at surgical operation in the spinal cord disease.

About the Author—Haiyun Li received his PhD degree in the Dept. of Biomedical engineering, Sun Yat-Sen University in 1997. In 2000/6-2002/10, he was Postdoctoral Fellow in vision and image processing Lab, National University of Singapore, Singapore. In 2008/10-2009/10, he was the visiting scholar, Henry H. Wheeler Jr. Brain Imaging Center, Helen Wills Neuroscience Institute, University of California, Berkeley. USA. Currently, he is a Professor in School of Biomedical Engineering, Capital Medical University, Beijing, China.His current research interests include Computer simulation and Medical Image Computing, MRI/fMRI, Medical system modelling and computing.
